# Microglia affect α-synuclein cell-to-cell transfer in a mouse model of Parkinson’s disease

**DOI:** 10.1186/s13024-019-0335-3

**Published:** 2019-08-16

**Authors:** Sonia George, Nolwen L. Rey, Trevor Tyson, Corinne Esquibel, Lindsay Meyerdirk, Emily Schulz, Steven Pierce, Amanda R. Burmeister, Zachary Madaj, Jennifer A. Steiner, Martha L. Escobar Galvis, Lena Brundin, Patrik Brundin

**Affiliations:** 1 Center for Neurodegenerative Science, Van Andel Research Institute, 333 Bostwick Ave., N.E., Grand Rapids, Michigan 49503 USA; 2 Laboratory of Neurodegenerative Diseases, Institut François Jacob, MIRCen, CEA, CNRS, 92265 Fontenay-aux-Roses, France; 3 Optical Imaging Core, Van Andel Research Institute, Grand Rapids, MI USA; 4 Bioinformatics and Biostatistics Core, Van Andel Research Institute, Grand Rapids, MI USA

**Keywords:** Parkinson’s disease, Alpha-synuclein, Prion-like, Microglia, Lipopolysaccharide, Interleukin-4

## Abstract

**Background:**

Cell-to-cell propagation of α-synuclein (α-syn) aggregates is thought to contribute to the pathogenesis of Parkinson’s disease (PD) and underlie the spread of α-syn neuropathology. Increased pro-inflammatory cytokine levels and activated microglia are present in PD and activated microglia can promote α-syn aggregation. However, it is unclear how microglia influence α-syn cell-to-cell transfer.

**Methods:**

We developed a clinically relevant mouse model to monitor α-syn prion-like propagation between cells; we transplanted wild-type mouse embryonic midbrain neurons into a mouse striatum overexpressing human α-syn (huα-syn) following adeno-associated viral injection into the substantia nigra. In this system, we depleted or activated microglial cells and determined the effects on the transfer of huα-syn from host nigrostriatal neurons into the implanted dopaminergic neurons, using the presence of huα-syn within the grafted cells as a readout.

**Results:**

First, we compared α-syn cell-to-cell transfer between host mice with a normal number of microglia to mice in which we had pharmacologically ablated 80% of the microglia from the grafted striatum. With fewer host microglia, we observed increased accumulation of huα-syn in grafted dopaminergic neurons. Second, we assessed the transfer of α-syn into grafted neurons in the context of microglia activated by one of two stimuli, lipopolysaccharide (LPS) or interleukin-4 (IL-4). LPS exposure led to a strong activation of microglial cells (as determined by microglia morphology, cytokine production and an upregulation in genes involved in the inflammatory response in the LPS-injected mice by RNA sequencing analysis). LPS-injected mice had significantly higher amounts of huα-syn in grafted neurons. In contrast, injection of IL-4 did not change the proportion of grafted dopamine neurons that contained huα-syn relative to controls. As expected, RNA sequencing analysis on striatal tissue revealed differential gene expression between LPS and IL-4-injected mice; with the genes upregulated in tissue from mice injected with LPS including several of those involved in an inflammatory response.

**Conclusions:**

The absence or the hyperstimulation of microglia affected α-syn transfer in the brain. Our results suggest that under resting, non-inflammatory conditions, microglia modulate the transfer of α-syn. Pharmacological regulation of neuroinflammation could represent a future avenue for limiting the spread of PD neuropathology.

**Electronic supplementary material:**

The online version of this article (10.1186/s13024-019-0335-3) contains supplementary material, which is available to authorized users.

## Background

In Parkinson’s disease (PD), misfolded α-synuclein (α-syn) assembles into intraneuronal Lewy bodies and Lewy neurites, the pathological hallmarks of this progressive neurodegenerative disorder. α-Syn is believed to play key roles in the pathogenesis of both idiopathic PD and some genetic forms of the disease [1]. In experimental models, α-syn assemblies can spread from cell to cell and from one brain region to another, which may explain the characteristic pattern of pathology identified in a majority of PD brains [2–6]. While this prion-like spread of pathology has been reproduced in many PD models [7, 8], little is known regarding how microglia influence this process.

Microglia represent the innate immune system in the brain and help to maintain homeostasis. Under resting conditions, microglia are dynamic cells that constantly survey their surroundings for infection and cellular distress [9]. They play a major role in the inflammatory process, as they rapidly respond to pathological insults, given their high phagocytic capacity [10, 11]. Microglial activation in response to injury and pathogens can lead to neuroinflammation, which, in turn, is known to contribute to worsening of neuropathology in animal models of PD [12]. An imaging study in PD patients identified activated microglia, notably in the basal ganglia and neocortex [13].

Microglia can display a wide range of activation states. Two main types of activated microglia have been defined: i) classically activated (cytotoxic) microglia that affect neuronal survival and that secrete pro-inflammatory cytokines such as tumor necrosis factor-α (TNF-α), interleukin-1β, interleukin-12, interferon-γ, or nitric oxide [14, 15]; and ii) alternatively activated (cytoprotective) microglia that support an anti-inflammatory response and prevent classical microglial activation. Alternatively activated microglia secrete interleukin-4 (IL-4), interleukin-13, or transforming growth factor β [14, 15]. Serum and cerebrospinal fluid from PD patients have a pro-inflammatory profile, suggesting cytotoxic microglial activity [16–18]. Microglia and misfolded α-syn are believed to participate in a vicious cycle: α-syn itself can cause an immune response, as both secreted and aggregated α-syn are known to activate microglia, and inflammatory mediators can promote α-syn aggregation [19–21]. Microglia are highly efficient at engulfing extracellular α-syn aggregates in vitro [22], but it is unknown whether resting-state microglia or if different forms of activated microglia affect the cell-to-cell transfer of α-syn in vivo [23].

The pro-inflammatory stimulus of lipopolysaccharide (LPS) induces microglia activation, which slows the degradation of α-syn within these stimulated microglia, leading to its accumulation in the cytoplasm in vitro [22]. It is possible that the disposal of extracellular α-syn by microglia is blocked when α-syn production coincides with, or is preceded by, inflammatory microglial activators such as exposure to LPS, bacteria, or environmental toxins [22]. In contrast, stimulation of human macrophages or microglia with IL-4, a well-described immune regulatory cytokine able to suppress inflammation [24], has been shown to enhance amyloid-β degradation in an in vitro model of Alzheimer’s disease [25, 26]. Intraperitoneal LPS injections prior to intravenous administration of recombinant α-syn pathogenic strains in wild type mice induce microgliosis and promotes recruitment of leukocytes to the central nervous system. α-Syn was internalized by LPS-primed inflammatory monocytes which were found within the brain, suggesting that LPS priming promoted α-syn spreading toward the brain [27].

Intrastriatal neural grafting is an experimental therapy that, in the best cases, has provided long-term relief of motor symptoms in some PD patients [28]. Notably, Lewy pathology has been documented in a small percentage of the grafted neurons of these patients [29–33]. It is unknown how Lewy pathology develops in naïve, embryonic cells but it is hypothesized that α-syn may transfer from the host brain to the grafted cells, seeding endogenous protein akin to the process in prion diseases [34].

We decided to use a model based on this clinical scenario and engineer a mouse brain to overexpress human α-syn (huα-syn) in the nigrostriatal pathway and then monitor cell-to-cell transfer of huα-syn from host nigrostriatal terminals to naïve dopaminergic neurons grafted into the striatum. The presence of α-syn in grafted dopaminergic neurons and within host microglia was quantified as the readout for α-syn cell-to-cell transfer. This study has two parts. The first hypothesis proposes that under normal conditions, microglia take up α-syn from the extracellular space, resulting in reduced α-syn transfer from neuron to neuron. To address this hypothesis, we used the selective colony stimulating factor 1 receptor (CSF1R) kinase inhibitor, PLX 5622, to deplete microglia numbers, in line with earlier studies [35–38]. We proposed that the relative absence of microglia would lead to more neuron-to-neuron α-syn transfer from the host striatum to naïve grafted neurons. Our second hypothesis is that different microglial phenotypes will either slow or accelerate neuron-to-neuron α-syn propagation. Specifically, we propose that IL-4-activated microglia effectively reduce the pool of extracellular α-syn and thereby decrease α-syn transfer from neuron to neuron. By contrast, we suggest that LPS-treated microglia are less prone to carry out phagocytosis [22] and therefore will be less effective at clearing α-syn.

## Methods and materials

### Animals

We purchased 10- to 14-week-old female C57BL/6 J mice as graft hosts, as well as male and female mice that were mated and used for embryo donations, from the Jackson Laboratory. Mice were housed at a maximum of five per cage under a 12-h light/12-h dark cycle with access to food and water ad libitum. The housing of the animals and all procedures were carried out in accordance with the *Guide for the Care and Use of Laboratory Animals* (United States National Institutes of Health) and were approved by the Van Andel Research Institute’s Institutional Animal Care and Use Committee.

### Vector preparation

We used an adeno-associated virus vector, AAV2/5, in which the expression of a human wild-type α-syn gene was driven by the synapsin 1 promoter and enhanced using a woodchuck hepatitis virus post-transcriptional regulatory element (WPRE). Vector production was as previously performed at the Lund University vector facility, Sweden, and described previously [39, 40]. Briefly, a transfer plasmid carrying AAV2 inverted terminal repeats coding for the human wild-type α-syn gene downstream to the synapsin 1 promoter was generated and transfected into human embryonic kidney 293 cells using the calcium phosphate method. The construct also included the packaging plasmid pDP6 encoding the AAV5 capsid proteins [41, 42]. The cells were lysed with 50 mM Tris, 150 mM NaCl, pH 8.4 buffer and by performing freeze–thaw cycles in a dry ice/ethanol bath. The crude lysates were purified first by ultracentrifugation (1.5 h at 350000 x *g* at 18 °C) in a discontinuous iodixanol gradient, and the virus-containing fractions were purified by ion-exchange chromatography using fast protein liquid chromatography. Genome copy titer was determined using real-time quantitative PCR (qRT-PCR). The genome copy titer used in the injections was 2.6 × 10^14 viral particles/μL.

### Stereotactic surgery

We performed all surgical procedures under general anesthesia using an isoflurane/oxygen mixture. Mice were placed in a stereotaxic frame (Kopf) and vector solutions were injected using a 10-μL Hamilton syringe fitted with a glass capillary (outer diameter of 100–200 nm). We injected 1 μL of AAV2/5-huα-syn virus at the following coordinates for the substantia nigra (SN, flat skull position, coordinates relative to bregma and dural surface): antero-posterior: − 2.8 mm, medio-lateral: − 1.1 mm, dorso-ventral: − 4.3 mm). The AAV2/5-huα-syn particles were infused unilaterally at a rate of 0.2 μL/min and the needle was left in place for an additional 3 min before it was slowly retracted at a rate of 1 mm/min. Three weeks following virus injection, 2 μL of either sterile PBS (control animals), LPS (1 μg), or IL-4 (10 ng) was injected unilaterally into the right striatum at the following coordinates (flat skull position, coordinates relative to bregma and dural surface): antero-posterior: + 1.0 mm, medio-lateral: − 2.0 mm, dorso-ventral: − 2.5, 3.0 mm). Two dorso-ventral sites were used to spread the 2 μL volume evenly throughout the mouse striatum.

### Grafting procedure

We dissected the ventral mesencephalon from embryonic-day-12 mice in cold HBSS-Ca^2+/^Mg^2+^ buffer (Invitrogen), similar to what was previously described [40]. We incubated the pieces in HBSS-Ca^2+/^Mg^2+^ containing 0.1% trypsin and 0.05% DNase for 20 min at 37 °C. After rinsing in HBSS-Ca^2+/^Mg^2+^, the tissue was mechanically dissociated into a cell suspension containing a mixture of single cells and small pieces of tissue. After centrifugation (180 x *g*, 5 min, 4 °C), the supernatant was removed and the volume was adjusted to give a suspension equivalent to one ventral mesencephalon per animal in HBSS-Ca^2+/^Mg^2+^. The cells were stored on ice during the transplantation procedure. Three weeks after injection of the AAV2/5-huα-syn vector, each mouse received a unilateral intrastriatal transplant (2 μL, equivalent to one ventral mesencephalon) using a 10 μL Hamilton syringe at the same striatal stereotaxic coordinates as above. The study altering the activation state of microglia was conducted in three separate cohorts for histological assessment, with a total of *n* = 20 controls, *n* = 20 LPS-injected, and *n* = 23 IL-4-injected mice, plus one additional cohort for biochemical assessment (MesoScale and western blotting; *n* = 10 controls, *n* = 12 LPS-injected, and *n* = 12 IL-4-injected mice).

### Gene expression in grafted tissue using qRT-PCR

The striatum, graft, and SN were microdissected in PBS, on a glass slide using a dissecting knife, from three free-floating sections per mouse. Tissue was from mice perfused and fixed with 4% PFA and sectioned at 30 μm on a freezing, sliding microtome. The three sections were pooled from two mice. Serially diluted AAV 2/5 huα-syn was used as a standard, the contralateral striatum as a negative control, and the ipsilateral striatum as a positive control. Fifty nanograms of RNA was extracted using the RecoverAll Total Nucleic Acid Isolation Kit (Life Technologies) according to the manufacturer’s instructions. cDNA was created using Revert Aid Kit (Thermo Fisher) according to the manufacturer’s instructions. Using CellsDirect One-Step qRT-PCR Kit (Invitrogen), the qRT-PCR was run using the following Taqman probes: WPRE forward, CCGTTGTCAGGCAACGTG; WPRE reverse, AGCTGACAGGTGGTGGCA, as used in [43]. WPRE is an enhanced element within the AAV plasmid. Primers to the housekeeping gene *Gapdh* were used. The probes designed to *Gapdh* were spanning intron 7 with forward: GGCATTGCTCTCAATGACAA and *Gapdh* reverse: ATGTAGGCCATGAGGTCCAC.

### Administration of the CSF1R inhibitor

The CSF1R inhibitor PLX 5622 (PLX) was kindly provided by Plexxikon and formulated in AIN-76A standard chow by Research Diets at 1200 mg/kg. The experiment was conducted twice, once for histological analysis (control chow *n* = 8–18 and PLX *n* = 8–10) and once for western blot analysis (control chow *n* = 6 and PLX *n* = 8). In both experiments, mice were fed on chow containing PLX or the control chow starting 2 weeks following vector injection, and they remained on the chow until the end of the study (for a total of 3 weeks).

### Western blotting

We selectively dissected the striatum on both sides of the brain, graft tissue, and the SN region. Briefly, mice were injected with sodium pentobarbital (130 mg/kg; Sigma) for euthanasia. Over ice, the brains were dissected and placed into a brain matrix with the ventral side up. The first blade was placed in the brain at the level of the optic chiasm, then second blade was placed 3 mm anterior to the first blade. This tissue was removed and further dissected on ice. The cortex was removed from the striatum and a rectangle around the graft tissue was cut and the graft tissue placed into a pre-weighed tube. The striatal hemispheres were divided into separate pre-weighed tubes (ipsilateral and contralateral). Finally, we dissected out the SN. A blade was placed at the level of the pons (− 5 mm from bregma) and another blade inserted 1 mm posterior. The ipsilateral and contralateral SN were separated and placed into pre-weighed tubes. The tissue from the striatum, graft and SN were used for cytokine analysis. The striatal tissue was also used for western blotting and RNA-seq analysis. Tissues were resuspended in modified radioimmunoprecipitation assay buffer containing protease and phosphatase inhibitors mixture (ThermoFisher Scientific), sonicated, and then centrifuged at 14,000 x *g* for 45 min at 4 °C; the supernatants were saved. Protein concentrations were estimated using a BCA kit (Thermo Scientific). Lysates were separated on 4–15% SDS-polyacrylamide electrophoresis gels (Bio-Rad). After the separation, proteins were transferred to a nitrocellulose membrane, and nonspecific binding sites were blocked by TBS with 5% bovine serum albumin (BSA) or skim milk followed by incubation with antibodies to Iba-1 (1:500, WAKO), human α-syn (huɑ-syn, 4B12, 1:1000, BioLegend), phosphorylated α-syn at serine 129 (pS129, 1:5000, Abcam), mannose receptor (1:1000, Abcam), NFκB p65 (1:300, Santa Cruz), or phospho-NFκB p65 (1:200, Santa Cruz), LC3B-1 (1:1000, Cell Signaling) or LAMP1 (1:1000, Abcam), p62 (1:1000, Abcam) followed by HRP-conjugated secondary antibody (1:2000, Cell Signaling). For the LPS and IL-4 study, a total of 6 controls, 4 LPS animals, and 4 IL-4 animals were used for western blotting. For the PLX study, 6 controls and 8 PLX-treated animals were used for western blotting. Signals were detected by chemiluminescence (Super Signal, Thermo Scientific) using a BioRad Imager. The western blot bands were quantified using ImageJ software (National Institutes of Health, USA).

### Cytokine analysis

Brain homogenates from mice injected with PBS, LPS, or IL-4 were collected and stored at − 80 °C until analysis. An extra control group of six mice that received only an AAV injection and graft were also included. Tissue was homogenized using a bath sonicator (Sonicator, Ultrasonic processor XL2020, 50% amplitude, 1 s on and off for a total of 4 min) in TRIS-based lysis buffer containing protease and phosphatase inhibitors (ThermoFisher Scientific) and samples spun at 22,600 x *g*, 40 min, at 4 °C, and supernatant collected and stored at - 80 °C until needed. Protein concentrations were determined using a BCA kit (Thermo Scientific). Analysis was performed using the V-PLEX Pro-inflammatory Panel 1 (mouse) kit from MesoScale Discovery (Rockville, MA, USA). The kit includes the following cytokines, with their lower limit of detection shown: IFN-γ, 0.04 pg/mL; IL-1β, 0.11 pg/mL; IL-2, 0.22 pg/mL; IL-4, 0.14 pg/mL; IL-5, 0.07 pg/mL; IL-6, 0.61 pg/mL; KC/GRO, 0.24 pg/mL; IL-10, 0.95 pg/mL; IL-12p70, 9.95 pg/mL; and TNF-α, 0.13 pg/mL. Samples with zero cytokine levels were assigned the value of the respective detection limit. Analysis of IFN-γ, KC/GRO, IL-10, and IL-12p70 were not included in the study. Calibration solutions and buffers were prepared as described by the kit. Supernatants were thawed and samples were prepared to a concentration of 100 μg in 50 μL of sample. Diluent 41 from the kit was added to make up the sample to 100 μL, and samples were loaded onto the reading plate, incubated, and washed according to the manufacturer’s protocol. The plate was then read on the MesoScale QuickPlex SQ120. Samples from 10 control, 12 LPS-injected, and 12 IL-4-injected mice were analyzed.

### Immunohistochemistry and imaging

Mice were anesthetized with sodium pentobarbital (130 mg/kg; Sigma) and perfused transcardially with 4% paraformaldehyde in 0.1 M phosphate buffer. Brains were removed, post-fixed for 24 h in 4% paraformaldehyde, placed in 30% sucrose in phosphate buffer, and stored at 4 °C until sectioning. Immunohistochemistry was performed on free-floating cryomicrotome-cut sections (30 μm in thickness) encompassing the SN and the striatum.

For immunohistochemistry, free-floating sections were washed with 0.1 M PBS (pH 7.4), incubated with 3% H_2_O_2_ for 20 min to quench endogenous peroxidase activity, and then were blocked for 1 h with 5% normal goat serum and 0.3% Triton X-100. Next, the sections were incubated overnight at room temperature with antibodies to tyrosine hydroxylase (TH; 1:1600, EMD Millipore), Iba-1 (1:500, WAKO), and specifically for huα-syn (Syn-211; 1:1000, Invitrogen). For TMEM119 immunohistochemistry, fresh frozen sections were first mounted onto slides and left to dry and then incubated overnight in a chloroform:alcohol (1:1) solution. Heat-mediated antigen retrieval using Tris-EDTA buffer with 0.05% Tween-20 (pH 9.0) for 20 min was used prior to incubation with the primary antibody (overnight at room temperature). Then, the sections were incubated with appropriate biotinylated secondary antibodies (goat anti-rabbit, goat anti-mouse, 1:500, Vector Laboratories) followed by incubation with Avidin/Biotin ABC reagent (Vector Laboratories). Immunolabeling was revealed using diaminobenzidine, which yielded a brown-colored stain visible in bright-field light microscopy. The sections were viewed under a Nikon Eclipse Ni-U microscope (Nikon); images were captured with a color Retiga Exi digital CCD camera (QImaging) using NIS Elements AR 4.00.08 software (Nikon) using the following objectives: 2x magnification (air immersion, 0.1 N.A.), 10x magnification (air immersion, 0.45 N.A.), 20x magnification (air immersion, 0.75 N.A.), and 60x magnification (oil immersion, 1.40 N.A.).

For multi-labeling immunofluorescence, the sections were blocked with 10% normal goat serum, 0.4% bovine serum albumin, and 0.3% Triton X-100 in PBS for 1 h at room temperature. Next, sections were incubated with antibodies to TH (1:1600, EMD Millipore), huα-syn (Syn-211, 1:1000, Invitrogen), Iba-1 (1:500, WAKO), and pS129 α-syn (1:10,000, Abcam). Appropriate secondary antibodies (Alexa Fluor 488, 594 and 680, Invitrogen) were used, followed by incubation with DAPI (nuclear stain).

### Stereological counting

We used a computer-assisted cell quantification program (StereoInvestigator, MBF Bioscience) coupled to a Nikon Eclipse Ni-U microscope (Nikon). We analyzed three graft-containing sections per animal spaced by 180 μm (section interval = 6) for 6–13 animals per group. Contours of the region were drawn at 10x magnification (air immersion, N.A. 0.45). Quantifications were performed at 60x (oil immersion, N.A. 1.40) using a counting frame of 70 μm × 70 μm, grid size set to 150 × 150 μm, with a guard zone of 2 μm, and dissector height set at 12 μm. Parameters were chosen to minimize the coefficient of error to ≤0.10. TH and Nissl positive neuronal counts were conducted using stereology. Contours of the region were drawn at 10x magnification (air immersion, N.A. 0.45). Quantifications were performed at 60x (oil immersion, N.A. 1.40) using a counting frame of 100 μm × 100 μm, grid size set to 200 × 200 μm, with a guard zone of 2 μm, and dissector height set at 12 μm. For the PLX and LPS, IL-4 study, we analyzed 5–7 SN-containing sections per animal spaced by 240 μm (section interval = 8) for 8–10 animals per group.

### Confocal imaging

A minimum of 10 randomly selected TH-immunoreactive neurons or Iba-1-immunoreactive cells per mouse were imaged using confocal microscopy. Each cell was three-dimensionally reconstructed to determine intracellular huα-syn immunoreactivity within the cell of interest. For the PLX study, a total of 22 mice were imaged, and for the comparison between control, LPS and IL-4, a total of 63 mice were imaged. Stacks of 14–20 μm (0.2 μm increments in z) of individual cells imaged at a resolution of 0.07 pixel/μm (1024 × 1024 pixel density) and were obtained using Nikon A1plus-RSi laser scanning confocal microscope system. The following solid state lasers were used for excitation: 403, 488, 561, and 640 nm; bandpass of the emission filters: 403, bandpass 425–475 and emission 402.9; 488, bandpass 500–550 and emission 488.1; 594, bandpass 570–620 and emission 561.4; and 680, bandpass 663–738 and emission 640.0. Images were captured using a 60x oil immersion, N. A 1.40 objective. Images and 3D renderings were acquired with NIS Elements AR 4.00.08 software (Nikon). For the supplementary 3D reconstructions, Imaris 3.0 (Bitplane) was used. Surfaces segmentation for the red, green and blue channels was performed.

### Unbiased quantification of α-syn cell-to-cell transfer

Using MATLAB (Mathworks) we determined the proportion of grafted TH-immunoreactive neurons and Iba-1-positive microglia that contain huα-syn. The red and green channels were collected as 12-bit monochrome (gray) images for each channel. The green channel was used to create a mask aimed at identification of the neuronal cell body. This was accomplished using an averaging filter with large dimensions and an appropriate threshold. Additional masks were created for the green and red channels using individual thresholds. The two masks were combined. and the resulting composite image displayed white pixels where the green and red exceeded their thresholds and were in the cell body of the neuron. The data are presented as the percentage of TH-immunoreactive neurons and Iba-1-positive microglia cells containing huα-syn.

### Quantification of Iba-1 cell density

The density of Iba-1-positive cells was quantified using NIS Elements AR 4.00.08 software (Nikon) in a blinded manner. Briefly, graft pictures were obtained on a Nikon Eclipse Ni-U microscope (Nikon); images were captured with a color Retiga Exi digital CCD camera (QImaging) using NIS Elements AR 4.00.08 software (Nikon) and 20x magnification (air immersion, 0.75 N.A., *n* = 3/animal/hemisphere), and a border of the graft and striatum was outlined. All the Iba-1-positive cells in the field of view that were within the border were counted using the NIS Elements AR 4.00.08 software (Nikon) using the count feature.

### Assessment of microglial morphology

Color (RGB) images of Iba-1-stained grafts and striatum were acquired at 60x magnification (oil immersion 1.40 N.A.) using a Nikon Eclipse Ni-U microscope (Nikon). A total of nine images/animal were analyzed; three images/from three sequential brain sections through the striatum/graft. Images from within the graft were taken to sample the dorsal, mid, and posterior parts of the graft within each section. In order to assess the morphology of microglia in the samples, RGB color images were processed by a custom MATLAB (Mathworks) script. Cell bodies and other large regions were segmented first. Dynamic thresholds were determined for both the blue intensity and saturation channels of the image. Upper and lower bounds of the thresholds were set to match the full width at half maximum of curves fit to the histograms of each channel. If the signal met the required criteria for either the blue channel or the saturation channel thresholds, it was selected for further analysis. Segmented regions were filtered to remove small (under 2000-pixel) areas and to remove objects that touched the borders of the image. The segmented areas were eroded to more accurately conform to cell bodies.

In order to appropriately segment long, thin cellular processes like those often associated with microglia, additional steps had to be taken. The saturation channel alone was used for this portion of the analysis because the addition of the blue channel did not change the segmentation result. The image was first blurred using a Gaussian filter. The resulting signal was squared and convolved with an edge detection kernel to amplify areas of desired signal. A threshold was used to maintain intensity values within 20% of maximum. Segmented regions were filtered as above to remove small (under 2000-pixel) areas and to remove objects that touched the borders of the image. Outlines of the segmented regions were created to simplify the objects to single lines without changing the structure of the region. Cellular processes that were not segmented in the first step were identified by subtracting the segmented cell bodies from the outlined regions. These processes were then dilated and added back to the cell body segmentation.

User input was required to select the segmented regions that represented microglia to be quantified. Only cells that appeared in focus, completely segmented, and fully contained in the sample slice were selected for further analysis. Area and perimeter were recorded for each segmented region that was selected. The ratio of area:perimeter (referred to as hydraulic radius) was calculated. The area:perimeter index was used as a measure for microglial activation. Because activated microglia are amoeboid in shape, these cells would have a large area and small perimeter, increasing the index score.

### RNA sequencing (RNA-seq) library construction and sequencing

Total RNA was extracted from brain homogenates from the ipsilateral striatum from control, LPS and IL-4 injected mice using the QIAshredder and RNeasy micro kit with on-column DNAse treatment (QIAGEN), according to manufacturer’s protocol. The samples used for RNA-seq were from the biochemistry cohort of animals, separate from the set of mice used for immunohistochemistry and confocal analysis. Quality was assessed by Bioanalyzer (Agilent Technologies) and samples with an RIN > 8 were used for library construction.

### Construction and sequencing of directional mRNA-seq libraries

Libraries were prepared by the Van Andel Genomics Core from 500 ng of total RNA using the KAPA RNA HyperPrep Kit with RiboseErase (v1.16) (Kapa Biosystems). RNA was sheared to 300–400 bp. Prior to PCR amplification, cDNA fragments were ligated to Bioo Scientific NEXTflex dual adapters (Bioo Scientific). Quality and quantity of the finished libraries were assessed using a combination of Agilent DNA High Sensitivity chip (Agilent Technologies, Inc.) and QuantiFluor® dsDNA System (Promega Corp). Individually indexed libraries were pooled and 50 bp, paired end sequencing was performed on an Illumina NovaSeq6000 sequencer using an SP 100 cycle kit (Illumina Inc). Base calling was done by Illumina RTA3 and output of NCS was demultiplexed and converted to FastQ format with Illumina Bcl2fastq v1.9.0.

### RNAseq analysis

#### Differential gene expression analysis

Following sequencing, paired-end fastq files were aligned to the GRCm38 primary assembly with STAR v2.5 [44]. Alignments (bam files) were converted to feature counts using HTSeq v0.6.0 referenced against the ENSEMBLE annotation of mm10: GRCm38.95.chr.gtf counting against the feature “exon”, grouped by “gene_id”, and using the strand parameter “reverse” (supplemental material). This set included exon locations for 54,752 genomic entities including pseudogenes, lncRNAs, and 21,949 protein coding genes. The resulting gene_id counts were normalized using edgeR (TMM) and tested for significant differential expression with Limma and Voom in R (v3.3.1) [45–47]. Prior to statistical testing, genes without more than 0.1 counts per million (CPM) in at least 3 samples were excluded (giving 24,227 genes). Three contrasts were made comparing each pairwise combination of LPS, IL-4, and PBS treatment groups for differential gene expression. A significance threshold of FDR = 0.05 was used with a secondary abs (log2FC) > 1 filter used to define the highest magnitude changes. After the PBS group was excluded from further analysis an additional minimum average expression filter of log2CPM > 0 in either the LPS or IL-4 groups was imposed. Expression values (log2CPM) for each sample are included as supplemental data. Some expression data was later converted to FPKM or z-scores for later visualization and comparison. Z-scores were calculated based on the mean and standard deviation of the IL-4 treatment group only.

#### Gene ontology analysis

Gene ontology enrichment analysis was done using String v.11.0 [48] against a background of the 15,938 minimally expressed genes. Genes set enrichment (GSEA, Broad, V3.0) [10.1073/pnas.0506580102] was also used to identify affected pathways with both unranked (log2CPM) and preranked (FDR) analysis. Included as supplemental figure are the highest results based on the unranked GSEA. Grouping of enriched biological pathways was done using the Cytoscape (V3.7.1) with the add-on Autoannotate [49] and c5.bp.v6.2.symbols.gmt. Finally, a subset of biological process gene sets were downloaded from Amigo2 (v2.5.12) for Fig. 6e.

#### Availability of data and material

All data generated during this study are included in this published article [and its supplementary information files] or have been deposited in NCBI’s Gene Expression Omnibus [50] and are accessible through GEO Series accession number GSE130683 (https://www.ncbi.nlm.nih.gov/geo/query/acc.cgi?acc=GSE130683).

### Statistics

The Plexxikon study was performed on 8–18 control and 8–10 PLX-treated animals for histological analysis and was repeated for biochemical assessment (3–6 control and 4–8 PLX). Microglial density and morphology were analyzed using two-factor ANOVA, with treatment and hemisphere as factors. The cell-to-cell transfer counts were analyzed using negative binomial mixed-effects regression and a false discovery rate adjustment on linear contrasts via the R package ‘lme4’ (v3.3.0) (http://lme4.r-forge.r-project.org/ and https://www.r-project.org/).

The experiments involving changing the activation state of microglia were conducted in three separate sessions with a total of 20 controls, 20 LPS-injected, and 23 IL-4-injected mice used for immunohistochemistry and cell-to-cell transfer analysis. For biochemical analysis, another cohort of 10 controls, 12 LPS-, and 12 IL-4-injected mice were used. Analysis via a linear mixed-effects model with a random intercept for experiment revealed that the results and effect estimates were negligibly sensitive to experimental differences. Microglial morphology, density and qRT-PCR data were analyzed using one-way ANOVA or non-parametric Kruskall–Wallis test. Cytokine profiling in the graft, striatum and SN were analyzed using a gamma mixed-effects model with random intercepts for each animal due to the distribution of the data. Western blotting data were analyzed using a standard linear mixed-effects regression with a random intercept for each individual, with treatment and hemisphere as factors comparing ipsilateral and contralateral hemispheres and using Tukey’s multiple comparison test as the post hoc analysis. The cell-to-cell transfer counts were analyzed using negative binomial mixed-effects regression and a Benjamini-Hochberg false discovery rate adjustment on linear contrasts for all the analysis via the R package ‘lme4’ (v3.3.0). We report means plus or minus standard error of the mean. A *p*-value of < 0.05 was taken as significant for all statistical tests. All tests were two-sided. Graphs were prepared using Prism 6.0, GraphPad.

## Results

### CSF1R inhibitor reduced microglia numbers

**Fig. 1:**
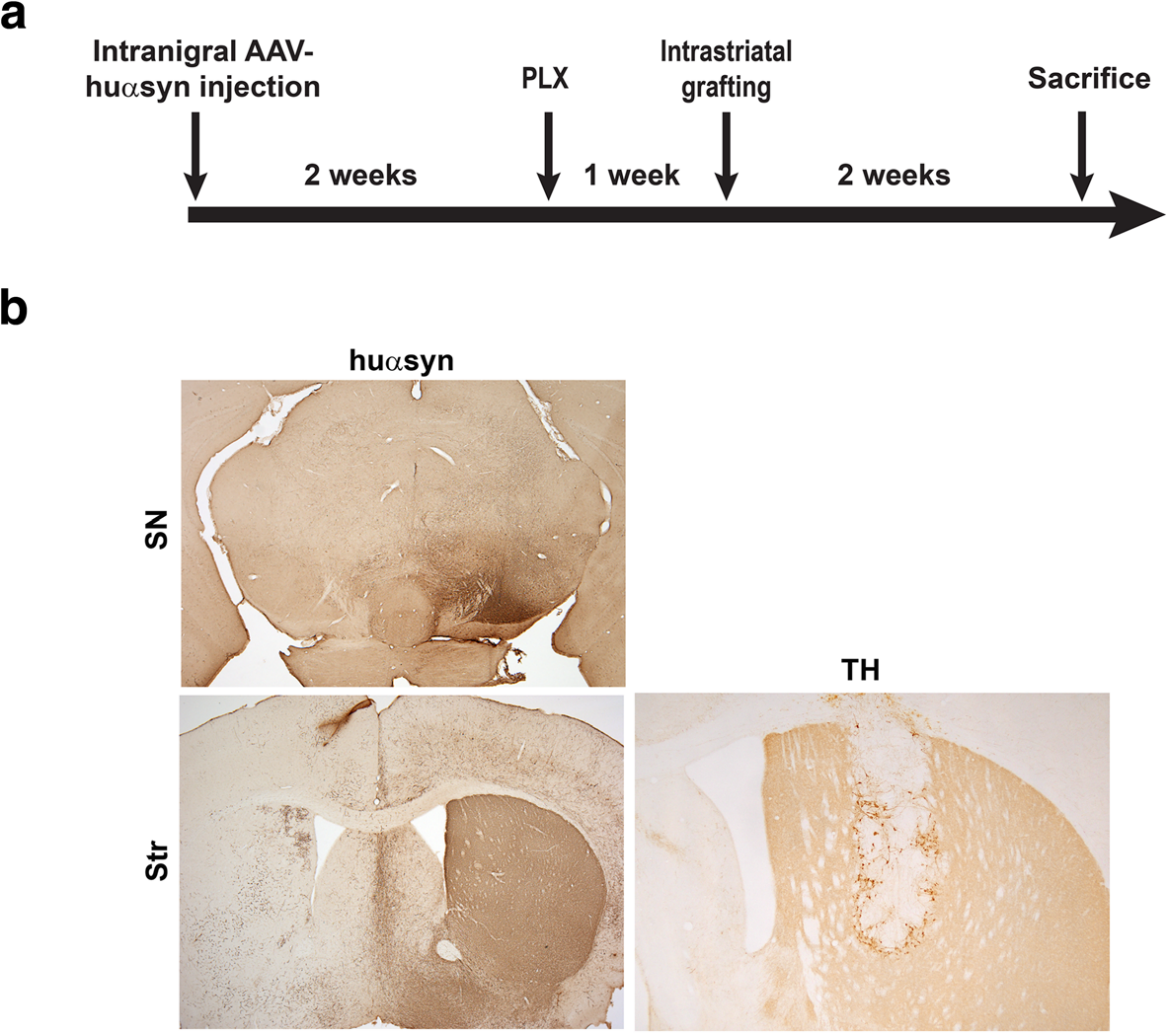
Elimination of microglia using a CSF1R inhibitor in a model of α-syn cell-to-cell transfer. **a** Experimental time line for the α-syn cell-to-cell transfer model. **b** Huα-syn expression in the SN and striatum by immunohistochemistry following unilateral stereotaxic injection in the SN. Surviving grafted neurons expressing TH within the striatum. The asterisk is in the center of the graft; SN; substantia nigra, Str; striatum, scale bars: 500 μm

We utilized a model of transplantation of embryonic mouse midbrain neurons into the striatum of a mouse overexpressing huα-syn via AAV2/5. Detection of huα-syn within the grafted naïve midbrain neurons that do not express huα-syn was our read-out for α-syn propagation from neuron-to-neuron. This model allows the manipulation of microglia by elimination or by promoting specific activation states. In this first study, we changed the number of microglia in an in vivo model of α-syn cell-to-cell transfer in order to understand the role microglia play in the propagation of α-syn pathology. A time line describing the study is shown in Fig. 1a. The expression of huα-syn in both the SN and striatum are represented in Fig. 1b; because the AAV2/5 vector was injected unilaterally, expression of huα-syn was only detected by immunohistochemistry in the injected hemisphere. When we confirmed the level of huα-syn expression by western blotting of striatal tissue (Additional file 1: Figure S1a, b), low levels of huα-syn expression were detected in the contralateral hemisphere (Additional file 1: Figure S1a, b). Presence of huα-syn in the contralateral side can be explained by crossed nigrostriatal projections; projections from the SN to the contralateral striatum have been documented [51, 52]. There was no evidence to suggest the reduction of microglia following PLX treatment significantly affected huα-syn expression (*p* > 0.05).

**Fig. 2:**
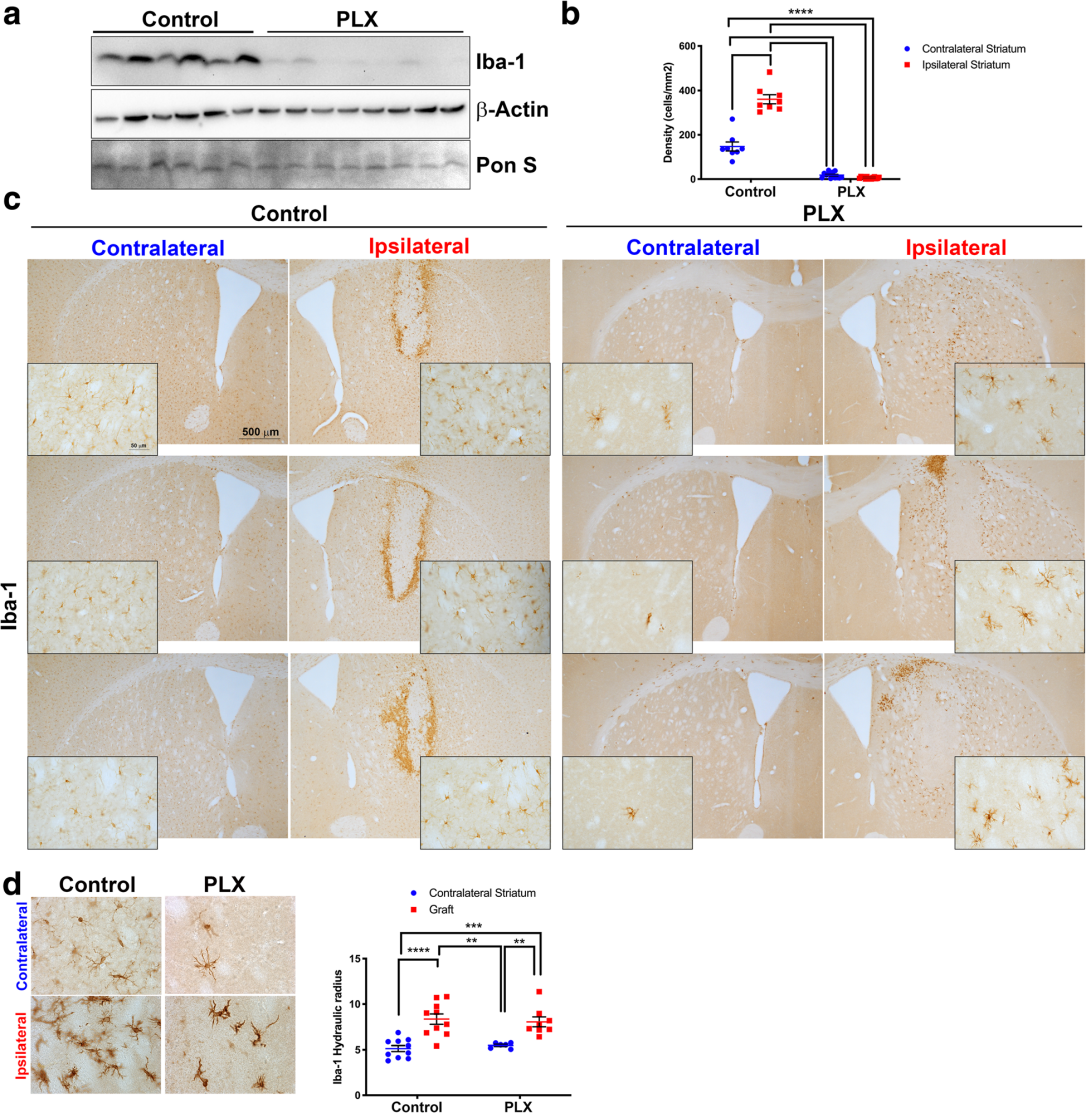
PLX treatment significantly decreases microglia numbers in the mouse striatum. **a** Microglia marker (Iba-1) protein as determined by western blotting shows significant depletion in striatal brain homogenates; *n* = 6 control, *n* = 8 PLX. **b** The density of microglia was reduced in striatal brain tissue stained for Iba-1. Quantification is shown above the images; *n* = 8 control, *n* = 8 PLX. ** = *p* < 0.01, *** = *p* < 0.001, **** = *p* < 0.0001. The error bars represent S.E.M. **c** Representative images of microglia through the striatum that contained the neural graft used for quantifying microglia density. The insert images are higher magnification taken from the same brain section; high magnification image scale bars = 500 μm; low magnification image scale bars = 50 μm**. d** Representative images of microglia morphology from the contralateral striatum and ipsilateral striatum. Quantification of microglia morphology (area/perimeter) is shown below the images; *n* = 10 control, *n* = 10 PLX. ** = *p* < 0.01, *** = *p* < 0.001, **** = *p* < 0.0001. The error bars represent S.E.M.

The microglial depletion experiment was conducted in two separate cohorts of mice, one for histological assessment of α-syn cell-to-cell transfer and one for biochemical assessment. Fresh-frozen tissue from mice was prepared for western blotting and probed with an antibody against Iba-1 that selectively recognizes microglia [53]. There was a significant reduction in the amount of Iba-1 in brain homogenates from mice fed the PLX chow (*n* = 8) relative to those from mice fed control chow (*n* = 6, Fig. 2a). To confirm that microglial were depleted in our model, we stained striatal brain sections for Iba-1 by immunohistochemistry. We found a significant reduction (80%) in the density of Iba-1-positive cells in mice fed PLX chow (Fig. 2b). We consistently observed that microglia were depleted throughout the striatum. We analyzed the density of microglia in sections that contained the neural graft and compared control and PLX-treated mice (Fig. 2c). The microglia that remained in the PLX-fed mice were located close the grafts. The difference in microglial cell numbers between the control and PLX-treated mice was pronounced in the contralateral hemisphere where no stereotactic injections took place (Fig. 2c). We further confirmed the reduction in microglia using an antibody directed towards the specific microglia marker TMEM119 [54] (Additional file 1: Figure S1c). PLX reduced the number of TMEM119-positive microglia in both the cortex and striatum compared to control mice. It has been reported that PLX compounds do not affect the morphology of the remaining microglia [55], and to confirm this in our model, we analyzed images of microglia from within the graft and striatum using a MATLAB script and calculated the hydraulic radius (area:perimeter ratio) of each cell. Activated microglia are amoeboid-shaped and therefore have a high index, i.e., a large area and a small perimeter. Our analysis revealed no significant difference in the hydraulic radius (area/perimeter) of Iba-1-positive cells between PLX-treated and control mice confirming that PLX did not significantly alter the morphology of remaining microglia (Fig. 2d). There were significant differences between the morphology of microglia in the contralateral striatum versus that of microglia located in and around the graft, most likely due to the stereotactic injections and grafting procedure. Collectively, our data show that the number of microglia can be significantly reduced using a CSF1R inhibitor (PLX 5622) in our mouse model of α-syn cell-to-cell transfer and that under the conditions used, the morphology of microglia did not differ between mice on control chow versus PLX chow.

Because autophagy is a pathway implicated in PD pathogenesis [56], we undertook further biochemical analysis of PLX-treated vs. control mice to explore whether PLX treatment altered autophagy markers or altered phosphorylated α-syn levels in these animals. Autophagy markers LC3B-1 and p62, as well as lysosomal marker LAMP1, were assessed using western blotting in control and PLX-treated mice. There were no significant differences between the control and PLX groups for the expression levels comparing LC3B-1, LAMP1 or p62 (Additional file 1: Figure S1a, b). There was no significant effect of PLX on the level of phosphorylation of mouse and huα-syn, as determined by using an antibody against pS129 α-syn (*p* > 0.05).

To confirm that AAV2/5 was not detected within the graft in our model, we performed qRT-PCR on dissected graft tissue. There were significant levels of viral particle present in the ipsilateral SN but not in the graft tissue samples (Additional file 2: Figure S2). Following confirmation that the virus was not present within the graft tissue, we identified grafted dopaminergic neurons by immunostaining for TH. Because the mouse striatum does not contain a significant number of TH-immunoreactive neuronal perikarya [57], the TH-expressing cell bodies that we identified are likely grafted neurons. We found that grafted dopaminergic neurons survived well in an environment of huα-syn overexpression as there was no significant cell loss of grafted dopaminergic neurons in the striatum (Fig. 1b, Additional file 3: Figure S3a). We also performed stereological cell counts of nigral neurons from control and PLX chow-fed mice and determined that AAV huα-syn nigral injections resulted in significant cell loss of both TH-positive and Nissl-positive neurons in our model (Additional file 3: Figure S3b).

### Microglia depletion increased accumulation of huα-syn

**Fig. 3:**
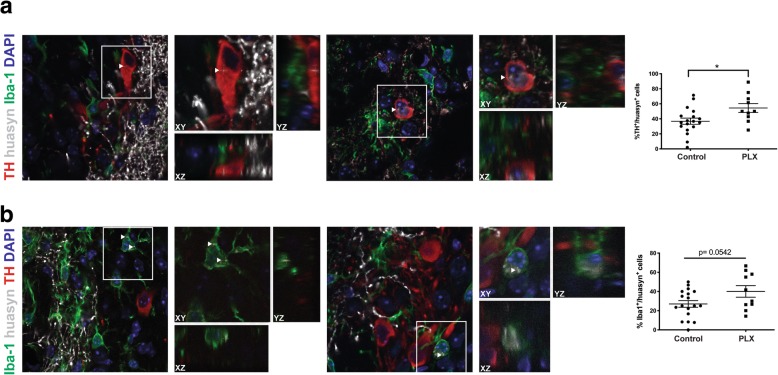
Fewer microglia following PLX treatment increased α-syn cell-to-cell transfer within the graft. **a** Confocal orthogonal reconstructions of grafted mouse TH-positive neurons (red) displaying intracellular puncta of transferred huα-syn (white); DAPI stain (blue) indicates nuclei. White box outlines cell of interest containing huα-syn puncta. The percentage of TH-positive neurons containing huα-syn. Control (circles) *n* = 18, PLX (squares) *n* = 10. * = *p* < 0.05. The error bars represent S.E.M. **b** Confocal 3D reconstructions of Iba-1 positive microglia (green), grafted mouse TH-positive neurons (red) displaying intracellular puncta of transferred huα-syn (white); DAPI stain (blue) indicates nuclei. White box outlines cell of interest containing huα-syn puncta. White arrows indicate huα-syn punctae. The percentage of Iba-1-positive microglia containing huα-syn. Control (circles) *n* = 18, PLX (squares) *n* = 10. The error bars represent S.E.M.

After establishing that PLX significantly reduced the number of Iba-1-positive cells within the striatum in our model, tissue sections were co-stained for Iba-1, TH, and huα-syn to quantify cell-to-cell transfer using confocal imaging. The α-syn 211 antibody was selected for this study due to its detection of huα-syn. Each confocal stack was analyzed using MATLAB to localize the huα-syn signal within the cell body. Because this antibody detects huα-syn and because there is no human protein in the grafted neurons that originates from mice, the source of the human protein within neurons and microglia is from the striatal terminals. Huα-syn was detected within grafted TH positive neurons and Iba-1 positive microglia (Fig. 3 and Additional file 4: Figure S4). There was a significant increase in the percentage of cells that were positive for TH and huα-syn in the PLX group versus control (Fig. 3a, *p* < 0.05), but the percentage of microglia containing huα-syn in the PLX group (relative to controls) was not statistically significantly different (Fig. 3b, *p* > 0.05).

### Differential activation of microglia affected α-syn cell-to-cell transfer

**Fig. 4:**
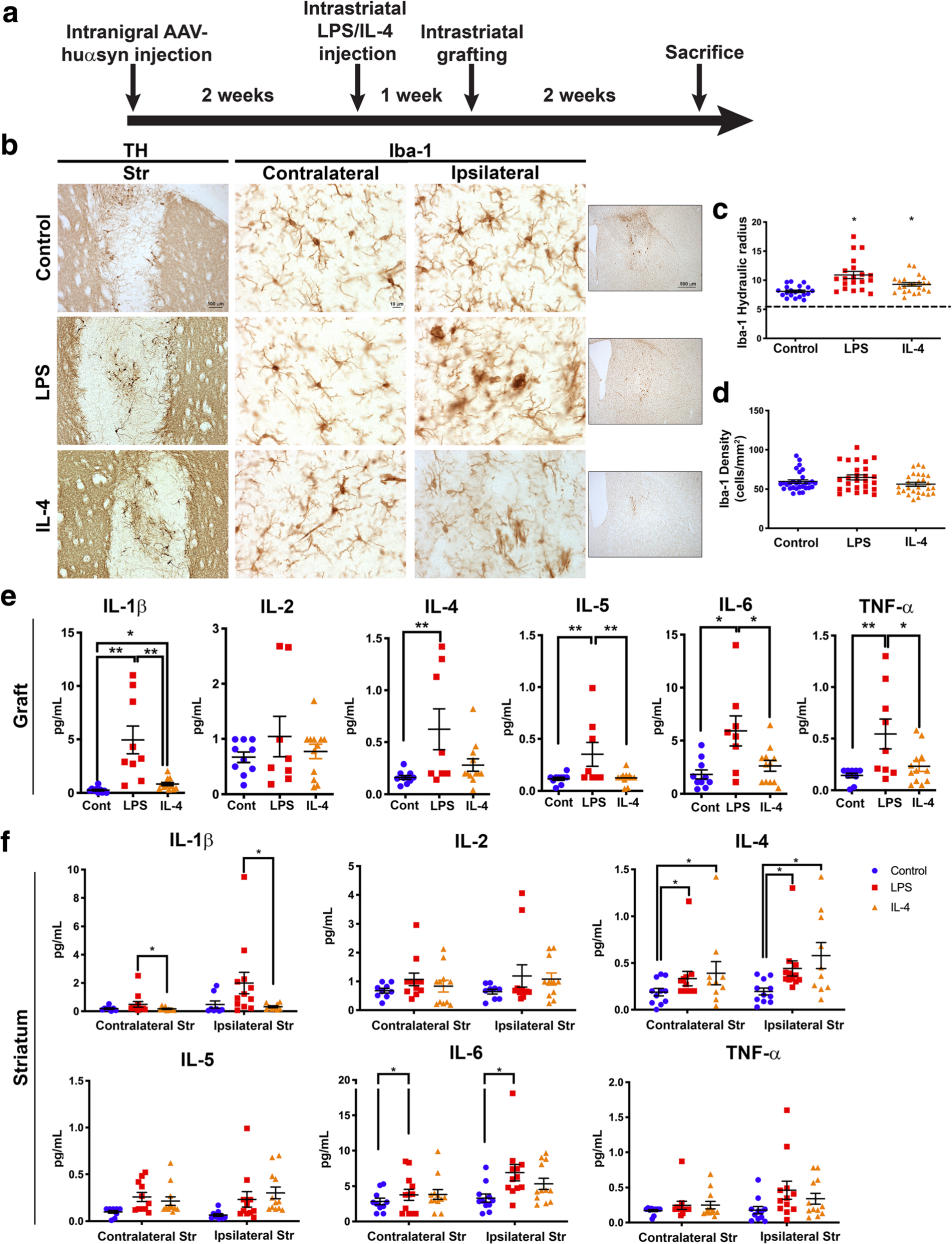
Altering the microglia activation state using a pro-inflammatory versus anti-inflammatory environment. **a** Experimental time line for the α-syn cell-to-cell transfer model. **b** Grafted neurons positive for TH survived all three experimental conditions (left column of images; scale bars, 100 μm). The contrast in microglial morphology using immunohistochemistry for Iba-1 between the contralateral and ipsilateral striatum (right two column of images). The inset images are of the ipsilateral striatum indicating microgliosis around the graft and where the larger images are taken from; scale bars, 10 μm and 500 μm **c** Ipsilateral microglial morphology as assessed by the area:perimeter index (hydraulic radius). The horizontal line represents the mean area:perimeter index score of microglia from the contralateral striatum. Microglia in the LPS group had significantly higher area:perimeter index than the control. Area:perimeter index was significantly different between control and IL-4 (control, *n* = 20; LPS, *n* = 20; IL-4, *n* = 23; *p* < 0.05). **d** There was no significant difference in the density of microglia from the ipsilateral striatum between control, LPS-, or IL-4-injected mice. **e** Inflammatory cytokines measured using the MesoScale pro-inflammatory panel 1 assay in graft tissue and **f** in striatal tissue; control, *n* = 10; LPS, *n* = 8–12; IL-4, *n* = 12. * = *p* < 0.05, ** = *p* < 0.01, *** = *p* < 0.001. The error bars represent S.E.M.

Given that a decrease in microglia led to an increase in the frequency of cells that exhibited punctae of huα-syn within TH-positive grafted neurons, we explored the effect of the activation status of microglia on neuron-to-neuron α-syn transfer. A time line of the paradigm used is shown in Fig. 4a. Immunohistochemistry was used to visualize the survival of grafted TH-positive neurons. Stereology counts of grafted neurons revealed that there was no significant evidence that the inflammatory status of the striatum influenced graft survival: the control (1499 ± 159), LPS-injected (1218 ± 142), and IL-4-injected (1097 ± 107) groups were not significantly different from each other (*p* > 0.05, Additional file 3: Figure S3c). We also performed stereological cell counts of nigral neurons from control, LPS-, and IL-4-injected mice and determined that AAV huα-syn nigral injections resulted in significant cell loss in our model, the median TH cell counts are: control; 2491, LPS; 2667 and IL-4; 2625 (Additional file 3: Figure S3d).

We also stained for Iba-1 to compare microglial morphology in both hemispheres (contralateral versus ipsilateral) within the striatum of control, LPS-, and IL-4-injected mice (Fig. 4b). We compared the morphology of Iba-1 immunoreactive microglia from the same anatomical level in the striatum in mice from each experimental group. Specifically, we used a MATLAB script to define the ratio between the perimeter and surface area of Iba-1 immunoreactive microglia within the graft, and this allowed us to calculate the hydraulic radius of each cell as an index of the activation state of the cell (Fig. 4c). Examples of the morphology of each group are found in Additional file 5: Figure S5a. Microglia within the graft were compared with cells from the contralateral striatum (Fig. 4c). The hydraulic radius was significantly increased in microglia from within the graft of LPS-injected mice, relative to controls (*p* < 0.05) and IL-4-injected mice were significantly different from controls (*p* < 0.05), indicating that microglia in both the LPS and IL-4 groups are activated. We also assessed the density of Iba-1-positive cells in the striatum of control, LPS- and IL-4-injected mice and found no significant differences between the groups (Fig. 4d). Overall, injections of LPS and IL-4 affected the morphology, but not the number, of microglia.

To further define the inflammatory environment in grafted mice from the control, LPS, or IL-4 groups, we operated on an additional cohort of mice — 10 control, 12 LPS-injected, and 12 IL-4-injected — and collected fresh tissue for biochemical analyses and followed the identical time line as previously used; see Fig. 4a for the time line of the experiment**.** At the conclusion of the experiment, we performed MesoScale immunoassays on dissected SN, striatum, and graft tissues (Fig. 4 and Additional file 6: Figure S6). As expected, the striatum and SN of mice injected with LPS or IL-4 were differentially affected compared to the controls. Specifically, we found an inflammatory response in the graft, striatum, and SN in LPS-injected mice (Fig. 4e and f; Additional file 6: Figure S6b) as shown by significantly elevated IL-1β, IL-4, IL-5, IL-6, and TNF-ɑ within the graft (relative to controls). There was no significant difference among the PBS control, LPS, or IL-4 groups in the IL-2 measured within the graft (Fig. 4e). The surrounding striatal tissue also had significant changes in inflammatory cytokines in the LPS and IL-4 groups compared with controls (Fig. 4f). Specifically, IL-6 level was significantly increased in LPS injected mice in both the contra- and ipsilateral hemispheres (*p* < 0.05). The level of IL-4 was significantly increased in LPS and IL-4 injected mice in both the contra- and ipsilateral striatum compared to controls (*p* < 0.05). Nigral tissue contained significantly increased levels of IL-1β, IL-2, IL-4, IL-5, IL-6 and TNF-ɑ in LPS injected mice compared to controls in both the contra- and ipsilateral SN (Additional file 6: Figure S6b). An additional group of mice that were AAV injected and only received a striatal graft, had below detection levels of IL-1β, IL-2, IL-4, IL-5, IL-6, and TNF-ɑ (data not shown).

The NF-κB signaling pathway, which typically is triggered following LPS stimulation [58], was up-regulated in the striatum of LPS-injected mice, relative to controls (Additional file 7: Figure S7a). Immunostaining for the mannose receptor (which mediates phagocytosis by microglia following IL-4 stimulation [59]) was significantly different between controls and IL-4-treated mice in the ipsilateral striatum (Additional file 7: Figure S7b). The levels of the mannose receptor were confirmed by confocal imaging of microglia from within the graft of injected mice (Additional file 7: Figure S7b). A summary of these findings is depicted in Additional file 7: Figure S7c.

### RNA-seq analysis in LPS compared to IL-4 striatal samples in a model of huα-syn cell-to-cell transfer

**Fig. 5:**
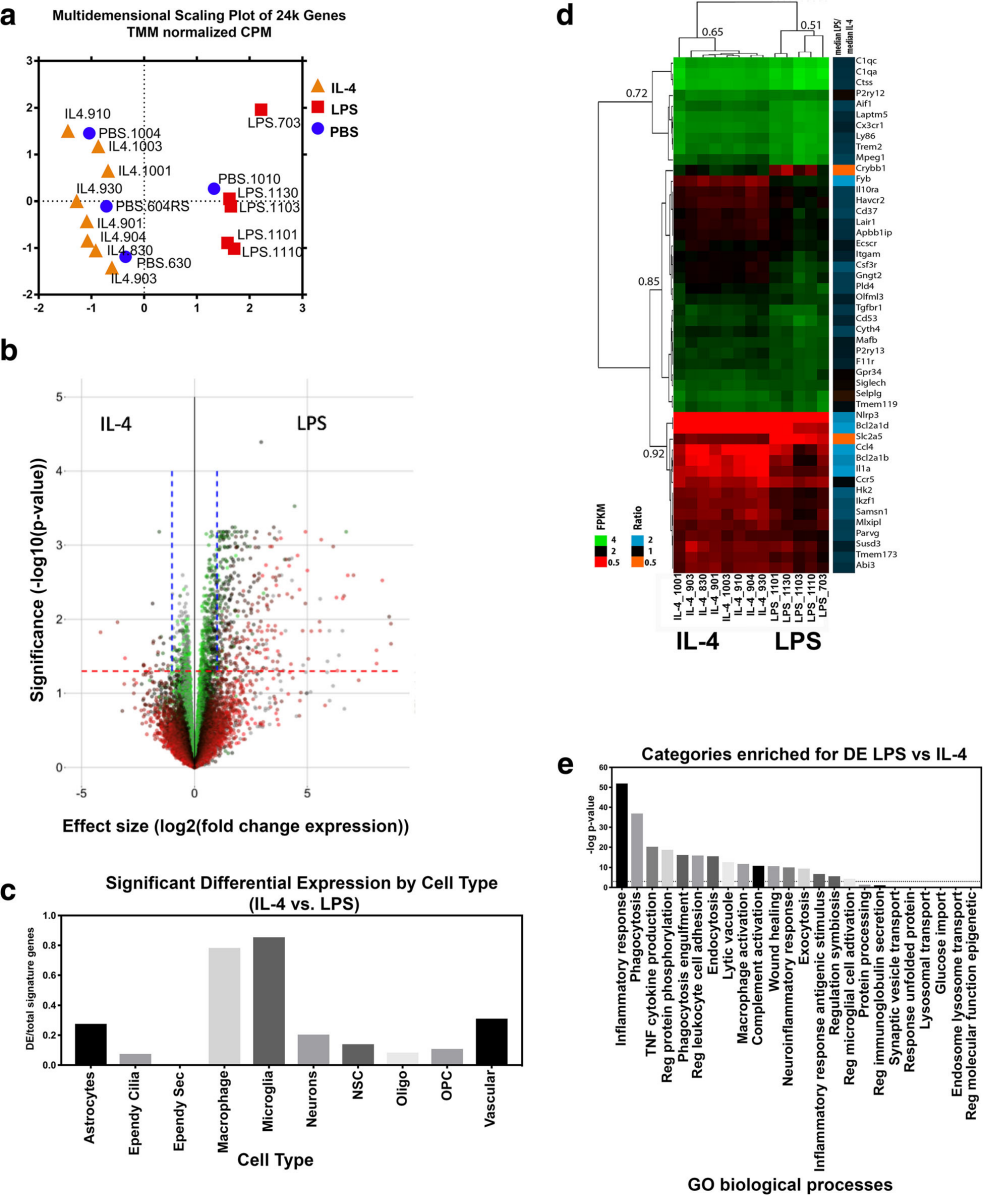
RNA seq analysis of pro-vs. anti-inflammatory environment in the model of αsyn cell-to-cell transfer. **a** MDS plot maintains distance between samples based on normalized CPM expression data for all mapped genes. **b** Volcano plot of RNA-Seq data where –log10(*p*-value) is plotted against the log2 fold change expression difference between LPS and IL-4 treatments for every mapped gene. The horizontal dotted red line corresponds to a FDR of 0.05 and the vertical dotted lines correspond to expression changes of 2-fold. Color corresponds to the median gene expression (CPM) in the PBS treatment group. **c** The top 50 signature genes were given for each of 10 cell types in Gokce et al. 2016. The ratio of genes for each cell type that are also significantly different (IL-4 vs. LPS: FDR < 0.05) are plotted. **d** Hierarchical clustering (eucledian distance, average linkage) of the RNA-seq results FPKM for the top 50 signature microglial genes with ratio of median LPS/median IL-4 expression. **e** Hypothesis driven gene ontology enrichment. A subset of biological process gene categories was selected. The enrichment significance measuring the overlap between gene members in these categories and significantly DE expressed genes is plotted. CPM; count per million, FDR; false discovery rate, FPKM; Fragments Per Kilobase Million, MSD; Multidimensional scaling

We performed RNA-seq using the dissected ipsilateral striatum from 4 PBS, 5 LPS and 8 IL-4 injected mice, overexpressing hu-αsyn as described above (Fig. 4a), to confirm the expected pro- vs. anti-inflammatory response associated with LPS vs. IL-4 injections, as quantified by the biochemical analysis above. The dimensional reduction technique, multidimensional scaling (MDS), indicated the overall variation in gene expression was closely tied to the LPS (pro-inflammatory) and IL-4 (anti-inflammatory) injection with a clear distinction between the LPS-, and IL-4-injected mice (Fig. 5a). The PBS injected mice samples grouped variously with either condition indicating an intermediate inflammatory state with high variation and further analysis focused on comparing IL-4 treated mice to the LPS treated mice. A total of 15,938 genes (including non-coding transcripts) were highly expressed in at least one of these two conditions and generated a measurement of at least 1 count per million (CPM) in either IL-4-, or LPS-injected mice these genes were further analyzed (Fig. 5b). Of these 15,938 genes, 792 were significantly differentially expressed [false discovery rate (FDR < 0.05)]. In addition, 217 genes showed a fold change of at least two (indicated by the blue dotted line on the volcano plot, Fig. 5b). Nearly all the expression changes were due to changes in the LPS-injected mice compared to the IL-4. Many of the identified differentially expressed genes had increased expression in the LPS-injected mice compared to IL-4 injected mice (Fig. 5b). Gene set enrichment (both quantitative and logistic) indicate that most altered pathways are related to inflammatory processes and these were activated in the LPS condition (Additional file 8: Figure S8a). Of the inflammatory processes that were activated in the LPS condition compared to the IL-4-injected mice, the adaptive immune response, migration and chemotaxis by immune cells and molecular activation of myeloid and dendritic cells were most prominent (Additional file 8: Figure S8b). These results indicated a pro-inflammatory profile in the LPS injected mice. When we compared our whole tissue differentially expressed (DE) genes to signature gene sets that define ten neuronal cell types (discovered using single-cell RNA-seq of mouse striatum by Gokce, et al., [60]) changes in macrophage and microglia markers were the most apparent in our data set, as predicted (Fig. 5c). As the predominant gene changes were observed in the macrophages and microglia cell types, we focused our analysis on specific microglial markers. The DE microglia markers were plotted in the heat map (Fig. 5d). A significant number of microglia markers were upregulated in the LPS-injected mice compared to the IL-4-injected mice, further illustrated by the increased median LPS/median IL-4 ratio (Fig. 5d). Overall, the primary categories enriched for in LPS vs. IL-4 comparisons relate to inflammatory response, TNF cytokine production and macrophage activation (Fig. 5e). The RNA-seq data point to significant and clear distinctions between the LPS-injected and IL-4-injected mice, and the gene differences relate to inflammatory processes. These results confirm that LPS and IL-4 injections generate the expected inflammatory responses in our model.

### Increased levels of huα-syn following LPS exposure

**Fig. 6:**
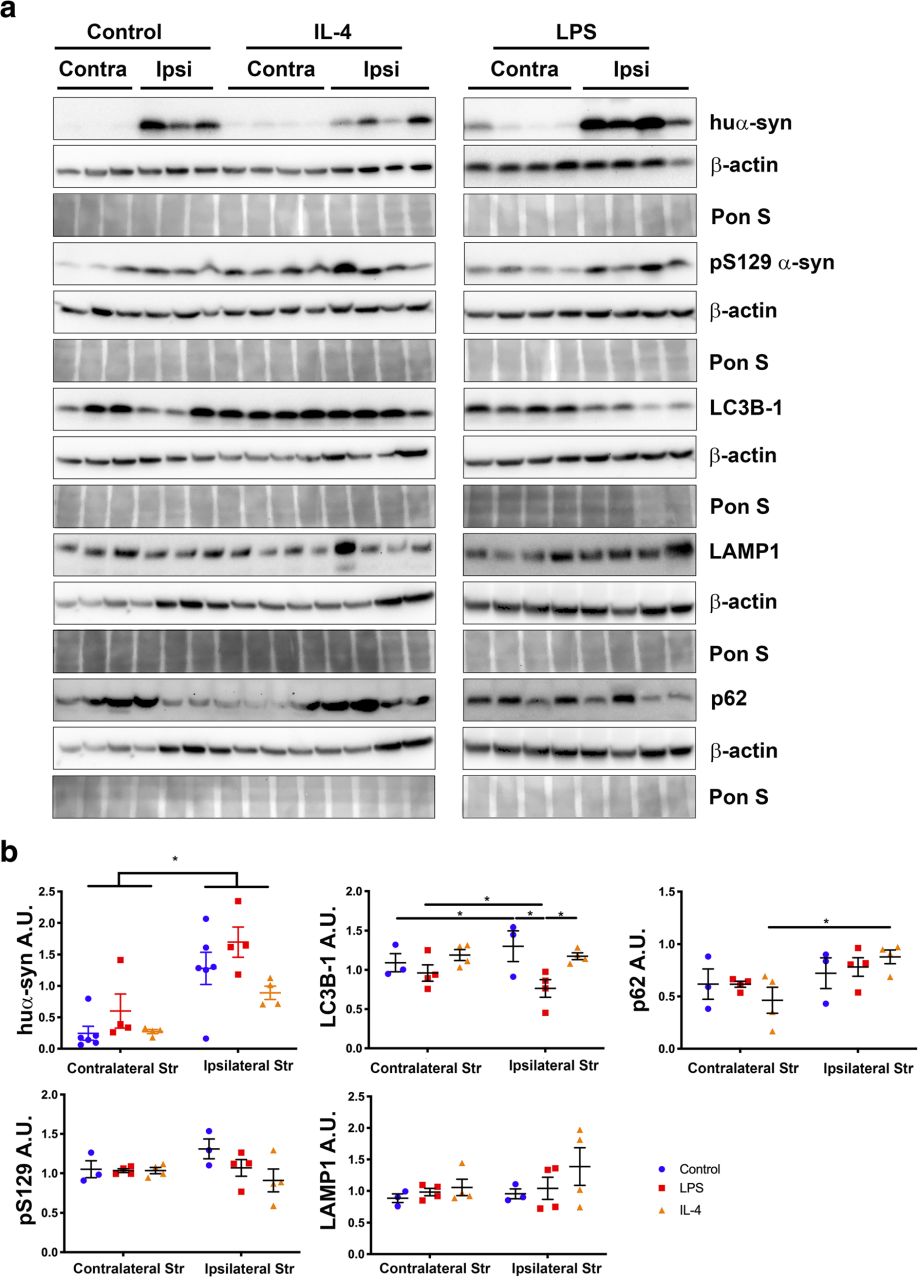
Increased huα-syn in the striatum of LPS-injected mice. **a** Western blots of striatal samples probed for huα-syn, pS129 α-syn, LC3B-1, LAMP1 and p62 in control, LPS-injected, and IL-4-injected mice. **b** Quantification of huα-syn, LC3B-1, and LAMP1 normalized to β-actin levels within the contralateral and ipsilateral Str of control, LPS-, and IL-4-injected mice. Pon S stain for loading reference. * = *p* < 0.05; error bars represent S.E.M. Pon S; Ponceau S, Str; striatum

As we confirmed the significant and contrasting inflammatory response to LPS and IL-4 by biochemical techniques and by RNA-seq, western blotting was used to establish whether LPS or IL-4 affected huα-syn protein amounts within the striatum (Fig. 6a). There was an overall significant effect of hemisphere, as the ipsilateral striatum had significantly higher levels of huα-syn than the contralateral striatum (*p* < 0.05). In neurodegenerative diseases such as PD, autophagy is impaired [56], and lipidation of LC3 1 to form LC3 II is part of autophagosome formation [61]. There was a statistically significant effect of hemisphere (*p* < 0.05) between contra- and ipsilateral striatum of control and LPS-injected mice for the levels of LC3B-1. There was also a significant decrease in LC3B-1 in LPS-injected mice compared to control and IL-4-injected mice in the ipsilateral hemisphere (Fig. 6b). There were no differences between hemispheres in the levels of p62, pS129 α-syn or LAMP1 (lysosomal membrane protein) [62] in control, LPS-, or IL-4-injected animals (Fig. 6b).

### Cell-to-cell transfer of huα-syn following LPS and IL-4 exposure

**Fig. 7:**
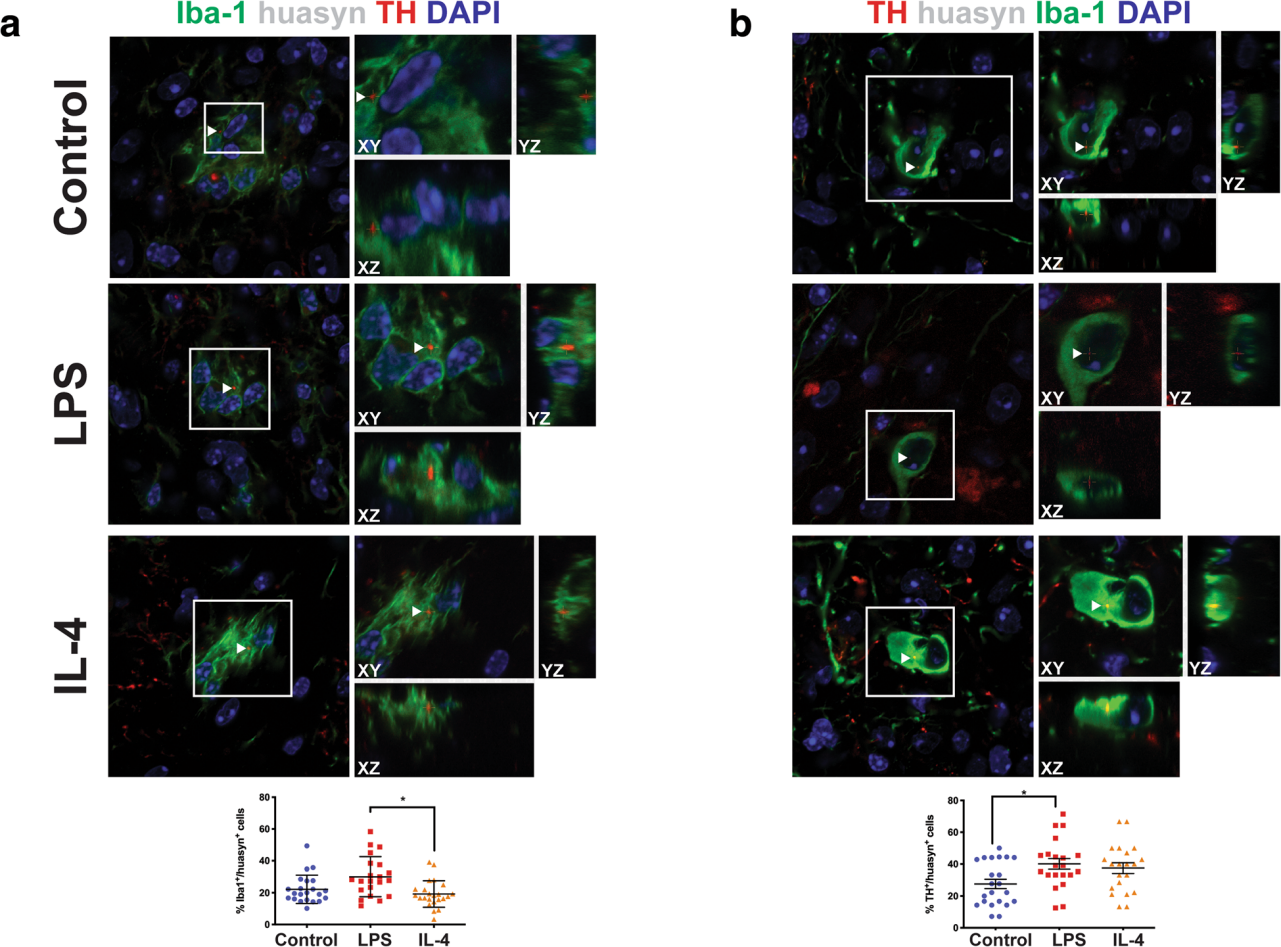
Cell-to-cell transfer of hu-αsyn into Iba-1-positive and TH-positive cells following microglia activation. **a** Orthogonal confocal reconstructions of microglial cells (Iba-1, green) containing intracellular puncta of hu-αsyn (red); nuclei are stained blue. White box outlines cell of interest containing huα-syn puncta. The percentage of Iba-1-positive microglia containing hu-αsyn (control, *n* = 20; LPS, *n* = 20; IL-4, *n* = 23). * = *p* < 0.05; error bars represent S.E.M. **b** Orthogonal confocal 3D reconstructions of TH-positive dopaminergic neurons (green) containing intracellular puncta of hu-αsyn (red); nuclei are stained blue. White box outlines cell of interest containing huα-syn puncta. White arrows indicate huα-syn punctae. The percentage of TH-positive neurons containing hu-αsyn (control, *n* = 20; LPS, *n* = 20; IL-4, *n* = 23). * = *p* < 0.05; error bars represent S.E.M.


Next, we quantified the cell-to-cell transfer of huα-syn by immunofluorescence and confocal imaging in mice injected with PBS (control), LPS, or IL-4 (Fig. 7). In our model, huα-syn was present within the graft, both in microglia and in grafted neurons (Fig. 7a, b, Additional file 9: Figure S9, Additional file 10: Video S1, Additional file 11: Video S2, Additional file 12: Video S3, and Additional file 13: Video S4). Using an antibody against pS129 α-syn (which detects both mouse and human phosphorylated α-syn), we observed pS129 α-syn within Iba-1-positive microglia (Additional file 5: Figure S5b). Exposure to IL-4 was predicted to increase the clearance of huα-syn by microglia, and the percentage of Iba-1 microglia that were positive for huα-syn was indeed significantly decreased in IL-4-injected mice relative to microglia in the LPS-injected group (Fig. 7a, *p* < 0.05). However, there was no significant difference in the proportion of Iba-1-positive microglia having huα-syn immunoreactive profiles, either between the IL-4 and control groups or between the LPS and control groups (Fig. 7a). We also analyzed the proportion of TH-positive grafted dopaminergic neurons that exhibited huα-syn immunostaining in the three experimental groups (Fig. 7b). In the LPS-exposed mice, the percentage of TH-positive cells containing huα-syn was significantly increased relative to controls (*p* < 0.05), but not relative to IL-4-injected mice. The results for the IL-4 and control mice did not significantly differ for this outcome parameter. Further evidence of cell-to-cell transfer of huα-syn to grafted neurons are provided in Additional file 10: Video S1 and Additional file 11: Video S2. Evidence of huα-syn within microglia are in Additional file 12: Video S3 and Additional file 13: Video S4. Additional files 11 and 13 are 3D renderings of a grafter neuron and of a Iba-1 positive microglia containing huα-syn puncta.

**Additional file 10:** **Video S1.** Confocal reconstruction of grafted neuron containing huα-syn punctae. Grafted neuron (green) containing huα-syn punctae (red, arrow) that is located within the neuron. DAPI stained the nucleus blue. (MP4 3559 kb)
**Additional file 11: Video S2.** 3D reconstruction of grafted neuron containing huα-syn punctae. Grafted neuron (green) containing huα-syn punctae (red) that is located within the neuron. DAPI stained the nucleus blue. (MP4 44689 kb)
**Additional file 12:** **Video S3.** Confocal reconstruction of Iba-1 positive microglia containing huα-syn punctae. Microglia (green) containing huα-syn punctae (red, arrow). DAPI stained the nucleus blue. (MP4 3007 kb)
**Additional file 13: Video S4.** 3D reconstruction of Iba-1 positive microglia containing huα-syn punctae. Two microglia cells (green) containing huα-syn punctae (red). DAPI stained the nucleus blue. (MP4 14119 kb)


## Discussion

Neuroinflammation is evident in PD and is believed to contribute to pathogenesis [16, 17, 63]. In addition, the spread of α-syn from cell to cell is believed to underpin the stereotypic anatomical patterns of α-syn aggregate pathology that have been described in PD. It is not known if microglia, as contributors to neuroinflammation, influence neuron-to-neuron spread of α-syn pathology in PD. In this study, we used a model that allows us to quantify in an unbiased manner the cell-to-cell transfer of huα-syn from the host striatum to naïve neurons grafted intrastriatally. By administering PLX, we reduced the number of microglia in the striatum by 80%. We found that the proportion of grafted dopaminergic neurons exhibiting huα-syn immunoreactivity increased significantly, indicating that microglia modulated the neuron-to-neuron transfer of α-syn in this experimental paradigm. When we used a similar transplant paradigm and altered microglia activation states by LPS or IL-4 injection, there was a significant reduction in the number of microglia containing huα-syn positive punctae under IL-4 treatment and a significant increase in the percentage of grafted neurons containing huα-syn punctae under LPS treatment. RNA-seq analysis confirmed that the inflammatory response from LPS-injected mice significantly differed from mice injected with IL-4 and that both the expression of inflammatory response pathway genes and of several microglia related genes were enriched in the LPS injected mice compared to the mice injected with IL-4. The inflammatory changes observed in the RNA-seq analysis were also reflected in the biochemical analysis we performed on the mouse striatum. These findings suggest that indeed, i) microglia play a role in α-syn cell-to-cell transfer and ii) anti-inflammatory microglia might enhance clearance of α-syn from the extracellular space.

### Reducing the number of microglia did not alter microglia morphology

We verified that chow delivery of the selective CSF1R kinase inhibitor PLX for 3 weeks results in an approximately 80% loss of microglia from the adult mouse CNS (see [36, 37, 64]). Notably, the morphology of the remaining microglia was unchanged, indicating that their activation status remained constant. While microglia morphology following PLX administration did not differ from the control condition in our experiments, we did not directly assess the phagocytic function of the remaining microglia. However, considering that microglial activation is characteristically associated with a change in morphology and that activated microglia are mainly scavenger/phagocytic cells [65], we believe that the microglia remaining after PLX treatment were quiescent.

### Reducing the number of microglia increased the proportion of grafted TH-positive neurons displaying huα-syn accumulation

Depletion of microglia has been shown to play an important function in homeostasis, neuroprotection and protein accumulation in mouse models of neurodegeneration [66]. In a different study, mice were infected with prion protein and given PLX 5622. The PLX 5622 treatment resulted in the expected reduction in microglial cell numbers and was associated with faster development of prion pathology and earlier death [67]. These results highlight that microglia are important for the removal of aggregated prion proteins. Interestingly, in models of Alzheimer’s disease with amyloid-β pathology, the depletion of microglia using PLX 5622 did not significantly impact amyloid-β plaques [35, 36]. These studies indicate that the role for microglia in modulating protein accumulation can be protein specific.

Although the increased proportion of grafted TH-positive neurons displaying huα-syn-immunoreactive puncta after PLX administration suggests that microglia are important for the clearance of huα-syn from the extracellular space, there are alternative explanations. For example, one might be the decreased degradation of huα-syn by the grafted neurons. With fewer microglia in the system, the increase in huα-syn taken up by neurons may be overwhelming. Neuronal degradation of monomeric wild-type α-syn occurs through the lysosomal pathways of chaperone-mediated autophagy and macroautophagy [68–70]. Excess α-syn has been shown to hamper efficient degradation by disrupting endoplasmic reticulum–to–Golgi trafficking of the transmembrane proteins involved in autophagosome biogenesis [71]. However, the levels of LC3B-1, LAMP1 and p62 did not change in our system, indicating that the observed effects could indeed be related to the depletion of microglia.

### LPS and IL-4 exposure influenced the presence of α-syn in microglia and grafted neurons

One focus of our study was to define the effects of LPS and IL-4 on microglia. All mice in our study were injected with an AAV vector to overexpress huα-syn in the nigrostriatal pathway, a paradigm which has previously been show to lead to microglial activation in rats [72]. We found that intrastriatal LPS and IL-4 injections produced the expected morphological changes in host microglia, and LPS exposure resulted in increased levels of the pro-inflammatory cytokines IL-1β, IL-5, and TNF-α, relative to IL-4 injected mice, both within the grafted tissue and in the surrounding host striatum. Interestingly, we also observed that IL-4 was significantly increased bilaterally in the striatum of both LPS- and IL-4-injected mice compared to controls. The increase in the LPS-injected mice could be due to production of IL-4 by microglia. Specifically, Bok et al., showed that rats injected with LPS in the SN had significant increases in IL-4 levels that remained elevated for 7 days post-LPS injection and that microglia were the source of IL-4 [73]. In addition, macrophages stimulated by LPS, both in vitro and in vivo*,* induced the expression of IL-4 and IL-5 genes via stimulation of TLR- 4 [74]. By biochemical analysis, we documented the inflammatory changes in our model and determined that LPS-injected mice produced a pro-inflammatory response compared to the IL-4 injected mice.

To further understand the microglia phenotype that is behind the changes in neuron-to-neuron transfer of α-syn, we conducted RNA-seq analysis comparing the ipsilateral striatum from LPS- and IL-4-injected mice. Gene ontology analysis revealed that there are several highly activated biological processes in the LPS group compared to IL-4 and the cell types most affected in our samples were microglia and macrophages. The biological processes significantly altered between LPS- and IL-4-injected mice related to the inflammatory response. This confirms that LPS-stimulated microglia were differentially stimulated compared to the IL-4 exposed microglia as determined by the mesoscale analysis. We further explored the nature of the inflammatory response by focusing on microglia-related genes. Specific microglia genes were upregulated in the LPS group compared to IL-4 group, including NLRP3 (NOD- LRR- and pyrin domain-containing 3) signaling receptor. This receptor is a cytosolic innate immune receptor ([75] Fig. 5d). NLRP3 activation is observed in the PD brain [76]. This implicates a pro-inflammatory process in our model consistent with increased levels of pro-inflammatory cytokines. Discrepancies between RNA-seq and protein levels in our model could be due to stability as mRNA are less stable than protein and as such, transcript levels partially predict protein levels [77]. Considering all the mice in this study were injected with an AAV vector to overexpress α-syn we can conclude that the LPS injection results in “over-activated” microglia [78].

Using western blotting we found that the level of huα-syn was significantly increased by approximately 50% in the striatum of LPS-treated relative to the IL-4-treated mice, which is consistent with work showing that an intranigral LPS injection promotes formation of aggregates of oxidized/nitrated α-syn in α-syn transgenic mice [79]. Furthermore, systemic LPS injections cause increased α-syn expression levels in the large intestine with the number of cells positive for α-syn were counted [80] and in the brain, LPS resulted in the formation of Lewy-like inclusions within nigral neurons [81]. Taken together, we conclude that an intrastriatal LPS injection creates a pro-inflammatory environment, in combination with increased amount of huα-syn, and that this is associated with increased huα-syn within host microglia in our hands.

Pro-inflammatory cytokines can also both be neurotoxic and attenuate microglial phagocytosis [82] which may play a role in our paradigm. LPS, the pro-inflammatory stimuli used in this study, was predicted to attenuate microglial phagocytosis [22] and lead to increased presence of huα-syn within microglia and grafted dopaminergic neurons. Indeed, we observed an increase in the proportion of dopaminergic neurons containing huα-syn. This observed phenomenon was not affected by cell death as the LPS or the IL-4 injections 7 days prior to grafting, affected the number of surviving grafted TH positive neurons. We hypothesized that altering the activation state of microglia would affect their ability to take up and process α-syn. Indeed, we observed that compared with microglia in LPS-injected mice, the proportion of microglia from IL-4-injected mice that exhibited α-syn was less. Following LPS injection, an increased proportion of grafted TH-immunoreactive neurons exhibited huα-syn, when compared to mice injected with PBS (control) or IL-4. Because we only followed the transplanted cells for 2 weeks after graft surgery, we did not expect to find protein-K resistant, aggregated α-syn. Earlier work has indicated that some aggregated α-syn can be detected in transplanted neurons 5 weeks after grafting [40, 83]. One reason to explain an increased number of TH neurons and microglia exhibiting huα-syn might be that the level of huα-syn was elevated within the striatum, as described above. Alternatively, dying nigral neurons expressing huα-syn could also lead to greater release of α-syn into the extracellular space [84]. However, the loss of nigral TH-immunoreactive neurons was similar in the LPS-injected, IL-4-injected, and control mice.

Our work suggests that microglia play multiple roles in neuron-to-neuron transfer of α-syn, presumably by modulating extracellular α-syn. From the PLX experiment, microglia appear to decrease α-syn neuron-to-neuron transfer. However, when microglia were activated using LPS, neuron-to-neuron transfer was increased, while IL-4 stimulated microglia were ineffective at significantly decreasing neuron-to-neuron of α-syn. We acknowledge that our model does not allow us to measure the dynamics of α-syn cell-to-cell transfer and define whether it is an increase in uptake and/or decrease in degradation that leads to the presence of α-syn within grafted neurons or microglia. A change in either uptake or degradation of α-syn in our model can perhaps explain why, in either the microglia depletion or the alternative activation of microglia scenario, we see an increased proportion of transplanted neurons containing huα-syn.

## Conclusions

Little is known about the factors that influence cell-to-cell transfer of α-syn in vivo. Our study demonstrates that the presence and activation state of microglia influenced this process in a neural transplantation paradigm. We found that a pro-inflammatory stimulus (LPS) enhanced the accumulation of huα-syn in grafted neurons. However, our study also suggests a physiologically protective role of microglia clearing α-syn, since removal of microglia also increased huα-syn accumulation in the grafted neurons. These observations contribute to our understanding of the interactions between the innate immune system and α-syn prion-like propagation.

## Additional files

Additional file 1: **Figure S1.** Reduction of microglia using the specific microglia marker TMEM119 and striatal levels of huα-syn, pS129 α-syn, LC3B, LAMP1 and p62 in control and PLX-treated mice. a Western blots of ipsilateral (I) and contralateral (C) striatal tissue from control and PLX-treated mice were probed for pS129 α-syn, huα-syn and autophagy makers LC3B-1, LAMP1 and p62 normalized to β-actin. Pon S for loading reference. b Quantification of panel a (control, circles, *n* = 3–6, PLX, squares, *n* = 4–8). Error bars represent S.E.M. Pon S; Ponceau S, Str; striatum. c Cortex and striatum images from control and PLX-treated mice indicating reduced TMEM 119-postivie microglia in PLX-treated mice, scale bar 50 μm. (TIF 138905 kb)

Additional file 2: **Figure S2.** Absence of AAV2/5 in grafted tissue. a Tissue sections and AAV standards were analyzed using qRT-PCR for AAV2/5 using the woodchuck hepatitis virus posttranscriptional regulatory element (WPRE) as a read out for viral particles, 5 weeks post injection. b The highest expression of virus was observed in the injected SN (triangles, *p* < 0.05), minimal expression was observed in the ipsilateral striatum and contralateral striatum, and no expression was observed in the graft; *n* = 3. Str; striatum, the error bars represent S.E.M. (TIF 101555 kb)

Additional file 3: **Figure S3.** Stereological counts of grafted after AAV injection. a There was no significant difference (*p* > 0.05) in the total number of TH-expressing cells in the striatal graft between control (*n* = 5, 2610 ± 942, circles) and PLX (*n* = 10, 1947 ± 432, squares) mice. b Significant loss in the total number of TH-expressing cells in the ipsilateral versus the contralateral SN in both control (circles) and PLX animals (squares, *p* > 0.05). Significant loss in the total number of Nissl-stained cells in the ipsilateral versus the contralateral SN in both control and PLX animals (*p* > 0.05) c There was no significant difference (*p* > 0.05) in the total number of TH-expressing cells in the striatal graft between animals in the control (*n* = 6, 1499 ± 159), LPS-injected (*n* = 13, 1218 ± 142), or IL-4-injected (*n* = 12, 1097 ± 107) groups. d Significant loss in the total number of TH-expressing cells in the ipsilateral versus the contralateral SN in control, LPS-, and IL-4 injected mice (*p* > 0.05). Significant loss in the total number of Nissl-stained cells in the ipsilateral versus the contralateral SN in control, LPS-, and IL-4 injected animals (*p* > 0.05) The error bars represent S.E.M. (TIF 102149 kb)

Additional file 4: **Figure S4.** Confocal reconstructions of grafted neurons and microglia from control and PLX-treated mice. Orthogonal views through multiple grafted neurons (red), Iba-1 positive microglia (green) and huα-syn (white) from control mice and PLX-treated mice indicating huα-syn within cross hairs. White arrows indicate huα-syn punctae. (TIF 131215 kb)

Additional file 5: **Figure S5**. Examples of microglial morphology and microglia containing pS129 α-syn. a Graft tissue from control, LPS-, and IL-4-injected mice representing morphological differences. Scale bars = 20 μm. b Microglia (Iba-1, green) from within the graft of control mice contain pS129 α-syn (white); nuclei are stained blue. (TIF 115252 kb)

Additional file 6: **Figure S6.** Example of tissue dissection and nigral cytokine profiling. a Fresh striatal tissue separating the graft (G) from the ipsilateral (Ipsi) and contralateral (Contra) striatum for cytokine profiling and western blotting. The graft tissue was cut in the shape of a rectangle using a blade to separate it from the striatum. b Nigral cytokine profiling within the contralateral and ipsilateral SN using the MesoScale assay on control (blue), LPS-injected (red), and IL-4-injected (orange) mice, ** = *p* < 0.01. Control, *n* = 8–10; LPS, *n* = 8; IL-4, *n* = 10–12; Str; striatum, error bars represent S.E.M. (TIF 106222 kb)

Additional file 7: **Figure S7.** Increases in pro-inflammatory signaling following LPS and IL-4 striatal injections. a Western blots of striatal tissue used to assess mannose receptor levels and NF-κB signaling in LPS- and IL-4-injected mice, normalized to β-actin. Pon S for loading reference. Quantification of contralateral and ipsilateral Str signals is shown below. * = *p* < 0.05; error bars represent S.E.M. b Confocal 3D reconstructions of mannose receptor signal (red) within microglia (Iba-1, green) from grafted control, LPS-, and IL-4-injected mice. Nuclei are stained blue and huα-syn is white; scale bars = 5 μm. c Summary of the LPS and IL-4 inflammatory profiles of injected mice. Pon S; Ponceau S, Str; striatum. (TIF 124784 kb)

Additional file 8: **Figure S8.** Enrichment of inflammatory processes. a The 217 DE genes (significant and with a change of expression of at least 2) are plotted as protein nodes (199 total) with edges indicating confidence of interaction or similarity. A total of 726 biological process categories are significantly enriched among this set of proteins, the most significant based on 103 proteins annotated as “immune system process” in red and 78 proteins annotated as “defense response” in green. b A cluster of the interrelated GO annotations based on the 726 enriched processes. Node size indicates the number of genes and edges represent overlap of protein members between ontologies. Automatic grouping is indicated by the orange circles with text summarizing GO categories and text size indicating the number of significant categories. c Top enrichment categories based on GSEA comparing LPS and IL-4 treated mice and using the expression values (log2CPM) for the 15,938 mapped genes with a minimum average expression of 1 CPM in either LPS or IL-4 treated mice. CPM; counts per million, GO; gene ontology, GSEA; gene set enrichment. (TIF 104231 kb)

Additional file 9: **Figure S9.** Confocal reconstructions of microglia and grafted neurons from control, LPS and IL-4 injected mice. Orthogonal views through multiple microglia (green) containing huα-syn punctae (red) and grafted neurons (green) containing and huα-syn (red) indicating huα-syn within cross hairs. White arrows indicate huα-syn punctae. (TIF 147289 kb)

## Data Availability

All data generated during this study are included in this published article [and its supplementary information files] or have been deposited in NCBI’s Gene Expression Omnibus [50] and are accessible through GEO Series accession number GSE130683 (https://www.ncbi.nlm.nih.gov/geo/query/acc.cgi?acc=GSE130683).

## Abbreviations

AAV: Adeno-associated virus
CPM: Count per million
CSF1R: Colony stimulating factor 1 receptor
DE: Differentially expressed
FDR: False discovery rate
GSEA: Gene set enrichment analysis
Iba-1: Ionized calcium-binding adapter molecule 1
IL-4: Interleukin-4
LPS: Lipopolysaccharide
MDS: Multidimensional scaling
NFκB: Nuclear factor kappa-light-chain-enhancer of activated B cells
NLRP3: NOD- LRR- and pyrin domain-containing 3
PCA: Principal component analysis
PD: Parkinson’s disease
PLX: Plexxikon 5622
pS129: α-syn phosphorylated on serine 129
RNA-seq: RNA sequencing
SN: Substantia nigra
TH: Tyrosine hydroxylase
TLR: Toll-like receptor
TNF-α: Tumor necrosis factor-α
α-syn: Alpha-synuclein
